# Histopathological Evaluation and Analysis of Immunohistochemical Expression of Bcl-2 Oncoprotein in Colorectal Carcinoma

**DOI:** 10.30699/ijp.2019.102982.2028

**Published:** 2019-09-22

**Authors:** Shilpa T Patil, Clement Wilfred D, Prasanna Shetty B

**Affiliations:** 1 *Department of Pathology, Vinayaka Missions Research Foundation, Karaikal, Pondicherry, India*; 2 *Department of Pathology, M.S. Ramaiah Medical College, Bangalore, India*

**Keywords:** Colorectal carcinomas, Immunohistochemistry, Prognostic factors

## Abstract

**Background & Objective:**

Colorectal cancer is the third most prevalent malignancy with high mortality rate, necessitating markers that predict survival and guide the treatment. Previous studies have examined the immunohistochemical expression of Bcl-2, an apoptotic marker, in colorectal carcinoma, but results have been contradictory. To evaluate the histopathological features of colorectal carcinoma, immunohistochemical expression of Bcl-2 must be analyzed to find out statistical association of Bcl-2 expression with certain prognostic factors histopathologic type, grade and TNM staging.

**Methods:**

This prospective study was conducted on the colectomy specimens of colorectal carcinoma, over a period of two years. The tumor morphology and Bcl-2 status were evaluated by immunohistochemistry in each case.

**Results:**

The study included 58 cases, with mean patient age of 47.07 years and male: female ratio of 1.89:1. Bcl-2 positivity was seen in 32.7% of the cases. Weak, moderate, and strong expression of Bcl-2 was seen in 12.1%, 12.1%, and 8.5% of cases respectively. Even though early stages of colorectal carcinoma showed greater frequency of Bcl-2 expression than advanced stages (36.3% versus 28%), however this association was not statistically significant.

**Conclusion:**

Lack of statistically significant correlation between Bcl-2 immuno-histochemical expression and prognostic parameters like tumor grade and stage, suggests that Bcl-2 immunoexpression may not be a significant prognostic marker in colorectal carcinoma.

## Introduction

Colorectal cancer (CRC) is the third most prevalent malignancy worldwide, with 6,63,904 new cases per year in men and 5,71,204 cases in women and is the second leading cause of death from malignancies in the industrialized nations (1, 2).

Results of surgical resection for advanced cancer are still poor, therefore the search for predictors of disease survival and identification of molecular markers is mandatory (3, 4). The most powerful predictors and guides to treatment in CRC are tumor stage and stage-independent factors including histologic grade, microscopic tumor type, vascular invasion, and surgical margins (1). Thus, meticulous histopathological examination of the CRC specimen is indispensable.

The pathophysiology of CRC is complex and the accumulation of molecular alterations, including Bcl-2 oncoprotein expression contributes to tumorigenesis (2, 5).

As Bcl-2 oncoprotein inhibits apoptosis and its over-expression contributes to neoplastic transformation, it has been studied for its potential impact on disease outcome (2, 5). Some clinical trials have associated Bcl-2 expression with favourable prognosis and others have shown no statistical correlation between Bcl-2 expression and prognostic factors like tumor stage and grade (2, 5). Thus, the objective of our study is to evaluate the histopathological features of CRC, and to investigate the association of Bcl-2 expression with prognostic parameters like tumor type, stage and grade. 

## Materials and Methods

**Source of Data**

This prospective study was conducted on colectomy, low anterior resection and abdominoperineal resection specimens of colorectal carcinoma, received in the Department of Pathology, for routine histopathological evaluation from the Departments of Surgery and Surgical Oncology, M S Ramaiah Medical College and Hospitals, Bengaluru, over a period of 2 years (from June 2015 to May 2017). Cases where only a biopsy, endoscopic mucosal resection or polypectomy has been performed and cases where there was extensive tumor necrosis without sufficient viable tumor cells were excluded from the study.

The specimens were received in 10% formalin. In every case, the standard protocol for surgical grossing of resected specimens was followed. After a detailed gross specimen examination, multiple representative tissue bits were taken from the tumor, surgical margins, mesentery, and all the lymph nodes. The latter were processed as per standard protocol and paraffin embedded tissue blocks were cut and stained by hematoxylin and eosin (H & E). The H & E stained slides were studied for the tumor histology and grade. The tumor was staged according to AJCC cancer staging system (6).

**Processing for Immunohistochemistry**

Immunohistochemical detection of Bcl-2 was done on 4µm thick sections, cut from a paraffin block of tumor tissue. The technique for IHC includes antigen retrieval in citrate buffer in a microwave oven, blocking endogenous peroxidase with 3% hydrogen peroxide, incubating with primary mouse anticlonal antibody against Bcl-2 (Anti-bcl-2 oncoprot), linking with rabbit anti mouse secondary antibody (Biogenex), enzyme labelling with streptavidin- horseradish peroxidase, developing chromogen with deaminobenzidine (DAB) and counterstaining with hematoxylin (7). Staining was defined as positive for Bcl-2 protein whenever any specific cytoplasmic staining was detected. In each case, the percentage of positive staining tumor cells (the number of positive tumor cells over the total number of tumor cells) was evaluated. A semi-quantitative assessment of staining was done as follows:

- Negative (0) – No Bcl-2 immunoreactivity detectable,
- Weak positive (1+) – less than 5% of tumor cells showing Bcl-2 positivity.
- Moderate positive (2+) - 5-50% of tumor cells showing Bcl-2 positivity.
- Strong positive (3+) - More than 50% of tumor cells positive for Bcl-2 (8).

**Statistical Analysis of Data:**

Descriptive statistics were employed to express quantitative parameters such as age, duration of the disease etc. and were summarized in terms of percentage with 95% confidence interval. Differences in the proportion of expression between different grades, types, etc. were tested for statistical significance by Chi-square test significance/ Fisher`s Exact test. Differences in mean values were tested by appropriate students` t`/ Mann-Whitney test.

## Results

During the study period, 58 resected specimens of colorectal carcinoma were received in the department of pathology. The Mean patient age was 47.07 years (age range: 25 to 75 years). Males (65.5%) were found to be highly susceptible to CRC when compared to females (34.5%) with a male to female ratio of 1.89:1. The most common site for colorectal carcinoma was sigmoid colon (37.9 %), followed by cecum (20.7%), descending colon (20.7%), ascending colon (6.9%) , hepatic flexure (6.9%), splenic flexure (3.4%), transverse colon (1.7%), and rectum (1.7%). The ulceroinfiltrative pattern was the dominant pattern seen in the gross specimen, accounting for 58.6%, followed by ulceroproliferative pattern (31.0%), annular constricting (5.2%) and diffuse infiltrating pattern (5.2%). Adenocarcinoma –NOS (ACa-NOS) (75.8%) was the predominant histologic type of colorectal carcinoma, followed by mucinous adenocarcinoma (MACa; 19.0%), and signet ring cell carcinoma (SCa; 5.2%). Majority of the tumors were grade 2 (moderately differentiated, 69%) followed by grade 1 (well differentiated; 17.2%), and grade 3 (poorly differentiated; 13.8%) morphology. Stage II (43.1%) was the most common presentation, followed by stage III (39.7%), stage I (13.8%), and stage IV (3.4%). Overall, 32.7% of CRC showed Bcl-2 positivity, out of which 20.6% of Bcl-2 expression was noted in left colonic tumors, 12.1% of Bcl-2 positivity was seen in right colonic tumors. Weak positivity, moderately intense positivity and strong positivity were observed in 12.1%, 12.1% and 8.5% of CRC cases respectively (Figures 1, 2, and 3 respectively & Table 1). Well and moderately differentiated CRC were associated with greater expression of Bcl-2 compared to poorly differentiated CRC (Table 2). Early stage (stages I and II) tumors showed greater expression of Bcl-2 in contrast to advanced stage tumors (stages III and IV) (Table 3). However, there was no statistically significant association between the Bcl-2 expression and tumor stage (*P*=0.5) and grade (*P*=0.58).

**Table 1:** Intensity of Bcl-2 in colorectal carcinoma

Intensity ofBcl-2	Frequency	(%)
0	39	**67.2**
1+	7	**12.1**
2+	7	**12.1**
3+	5	**8.5**
Total	58	**100.0**

**Table 2:** Bcl-2 Expression with respect to histologic grade

Bcl-2 expression	Histologic grade		
	G1 (%)	G2 (%)	**G3 (%)**
0	20.5	61.5	**17.9**
1+	14.3	85.7	**0.0**
2+	14.3	85.7	**0.0**
3+	0	80	**20**
Total	17.2	68.9	**13.7**

G1- well differentiated, G2- moderately differentiated, G3- poorly differentiated

**Table 3:** Bcl-2 expression with respect to tumor stage

Stage	0 (%)	1+ (%)	2+ (%)	3+ (%)
I	50	25	25	**0**
II	68	20	0	**12**
III	78.2	0	13.2	**8.6**
IV	0	0	100	**0**
Total	67.2	12.1	12.1	**8.6**

P-value =0.502 (*P*<0.05 is considered statistically significant).

0 – Negative, 1+, 2+, 3+ = weak, moderate and strong Bcl-2 expression respectively

**Table 4:** Comparison of Bcl-2 expression and its prognostic value in various studies

Studies	Sample size	Bcl-2 expression (%)	Prognostic value of Bcl-2	P-value
Pity IS et al.^9^	52	9.6	Low Bcl-2 expression was observed with advanced tumor stage; however this association didn’t reach the level of significance.	0.27
Ofner D et al.^10^	104	47.1	Bcl-2 oncoprotein appears to be associated with favourable clinical outcome since its expression decreased with increasing stage.	0.001
Manne U et al.^11^	134	41	Low Bcl-2 expression was significantly associated with higher tumor stage and showed favourable prognosis.	0.025
Zhao et al.^12^	93	57	Bcl-2 expression had no prognostic significance	>0.05
Contu PC et al.^13^	132	29.5	No significant association between bcl-2 and stage, despite a trend showing decreased bcl-2 expression among poorly and moderately differentiated tumours.	0.14
Ghita et al.^14^	22	18.18	High Bcl-2 expression was observed in early stages, which could represent an explanation for the better prognosis of these cases. However this association didn’t reach the level of significance	0.17
Current study	58	32.7	No statistically significant prognostic correlation between Bcl-2 and stage was present	0.502

P-value<0.05 considered statistically significant.

**Table 5:** Bcl-2 Expression with respect to histologic type

Bcl-2 expression	Histologic type		
	ACa-NOS (%)	MACa (%)	**SCa (%)**
0	69.2	25.6	**5.1**
1+	85.7	14.3	**0**
2+	100	0	**0**
3+	80	0	**20**
Total	75.8	19	**5.2**

ACa-NOS – Conventional adenocarcinoma, MACa- Mucinous adenocarcinoma, SCa- Signet ring cell adenocarcinoma

## Discussion

In the current study, Bcl-2 marker was targeted for immunohistochemical analysis for its role in cancer prognosis. Bcl-2 family members play important roles in tumor initiation and progression. Immunohistochemical expression of Bcl-2 was found in 32.7% of cases. The prevalence of Bcl-2 immunohistochemical expression in CRC varies greatly from one study to another (9-14). These conflicting data are partly due to different populations, different scoring systems, different statistical analyses, as well as primary factors regarding the immunohistochemical technique and the evaluation of the results.

**Intensity of Bcl-2 Expression:**

Intensity of Bcl-2 expression in our study differs from other studies due to varying sample sizes and different scoring system. Other studies have used different scoring systems, which makes the comparison between the studies difficult (15, 16).

**Bcl-2 and Histopathologic Type:**

In the present study, Bcl-2 positivity was found mainly in ACa-NOS compared to MACa, SCa. No statistical correlation between Bcl-2 positivity and histopathologic type was found (*P*>0.05). Qasim *et al*. found statistical significance between Bcl-2 and non-mucinous histopathologic type (16). Some studies have demonstrated Bcl-2 positivity in more than 30% of a mucinous adenocarcinoma (17). 

**Bcl-2 Expression and Histologic Grade:**

In the current study, well differentiated and moderately differentiated cases had greater Bcl-2 expression than the poorly differentiated cases, which was not statistically significant. One author had demonstrated that Bcl-2 overexpression seems to be associated with advanced histologic grade, resulting in a more aggressive tumor (15). Some studies had failed to demonstrate any correlation between Bcl-2 expression and histologic grade, in agreement with our studies (16). Petrisor *et al.* demonstrated that the proportion of Bcl-2(+) expression in poorly differentiated lesions is significantly lower than that in the other grades, which could explain a better prognosis for Bcl-2 positive cancers (18). 

**Bcl-2 Expression and TNM Stage:**

In the present study, Bcl-2 was expressed mainly in lower stages compared to advanced stages. There was no statistically significant correlation between the Bcl-2 expression and tumor stage. This finding was also demonstrated by many other studies (19, 20). One study has reported increased proportion of Bcl-2 expression in adenomas than in carcinomas, indicating the role of Bcl-2 in early neoplastic transformation (18). Another study had demonstrated statistically significant correlation with reducing Bcl-2 expression and increasing stage and poorer clinical outcome (21). Results concerning the role of Bcl-2 in relation to stage and survival are conflicting.

## Conclusion

In the present study, Bcl-2 positivity was expressed in 32.7% of CRC cases. Even though well and moderately differentiated CRC were associated with a greater expression of Bcl-2 compared to poorly differentiated CRC, this association was not statistically significant. Similarly, even though early stage tumors (stages I and II) were associated with greater expression of Bcl-2 than advanced stage tumors (stages III and IV), this association was not statistically significant. This lack of statistically significant correlation between Bcl-2 immunohistochemical expression and prognostic parameters like tumor grade and stage, suggests that Bcl-2 immunoexpression may not be a significant prognostic marker in CRC. However, follow up studies, with correlation between Bcl-2 expression and cancer specific 5-year survival statistics, need to be done for definite assessment of the prognostic value of Bcl-2 in CRC.

## References

[1] Vasile L, Olaru A, Munteanu M, Plesea IE, Surlin V, Tudorascu C (2012). Prognosis of colorectal cancer: clinical, pathological and therapeutic correlation. Rom J Morphol Embryol.

[2] Hegazy A, Daoud SA, Ibrahim WS, El-Atrebi K, Saker M, Abdel-Wahab N (2014). Role of Ki- 67, p53 and Bcl-2 in Advanced colorectal carcinoma (Histopathological and Immunohistochemical study). Academic J Cancer Res.

[3] Papagiorgis PC, Zizi AE, Tseleni S, Oikonomakis IN, Sofras L, Patsouris E (2012). Disparate clinicopathological correlations of p53 and Bcl-2 in colorectal cancer. Mol Med Rep.

[4] Luderer LA, Lustosa SAS, Silva SEM, Denadai MVA, AfonsoJr RJ, Viana LS (2015). Significance of a Biomarkers Immunohistochemistry Panel for Survival Prognostic in Patients with Sporadic colorectal cancer. Ann Clin Pathol.

[5] Menezes HL, Juca MJ, Gomes EG, Nunes BL, Costa HO, Matos D (2010). Analysis of the immunohistochemical expressions of p53, Bcl-2 and Ki-67 in colorectal adenocarcinoma and their correlation with the prognostic factors. Arq Gastroenterol.

[6] Edge SB, Byrd DR, Compton CC, Fritz AC, Greene FL, TrottiA (2009). AJCC Cancer Staging Manual.

[7] Bancroft JD, Gamble M (2008). Theory and practice of histological techniques.

[8] Al-Temimi SMA (2011). Correlation between Bcl-2 protein expression and clinicopathological parameters of colorectal carcinoma. Kufa Med Journal.

[9] Pity IS, Arif SH, Hadji DA (2013). Angiogenesis, p53 and Bcl2 in Colorectal Carcinoma. Int. J. Adv. Res. Technol.

[10] Ofner D, Riehemann K, Maier H, Riedmann B, Nehoda H, Totsch M (1995). Immunohistochemically detectable bcl-2 expression in colorectal carcinoma: correlation with tumour stage and patient survival. Br J Cancer.

[11] Manne U, Weiss HL, Grizzle WE (2000). Bcl-2 expression is associated with improved prognosis in patients with distal colorectal adenocarcinomas. Int J Cancer.

[12] Zhao DP, Ding XW, Peng JP, Zheng YX, Zhang SZ (2005). Prognostic significance of bcl-2 and p53 expression in colorectal carcinoma. Journal of Zhejiang University-Science B.

[13] Contu PC, Contu SS, Moreira LF (2006). Bcl-2 expression in rectal cancer. Arquivos de gastroenterologia.

[14] Ghiţă C, Vîlcea ID, Dumitrescu M, Vîlcea AM, Mirea CS, AşchieMA (2012). The prognostic value of the immunohistochemical aspects of tumor suppressor genes p53, bcl-2, PTEN and nuclear proliferative antigen Ki-67 in resected colorectal carcinoma. Rom J MorpholEmbryol.

[15] BHA Maather (2011). Immunohistochemical study of CEA and BCL2 Expression in Colorectal Adenocarcinoma and its Correlation with Some Pathological Parameters. Medical journal of Babylon.

[16] Qasim B, Ali H, Hussein A (2012). Immunohistochemical expression of p53 and Bcl-2 in colorectal adenomas and carcinomas using automated cellular imaging system. Iranian Journal of Pathology.

[17] Melincovici CS, Mihu CM, Mărginean MA, Boşca AB, Coneac A, Moldovan I (2016). The prognostic significance of p53, Bax, Bcl-2 and cyclin E protein overexpression in colon cancer-an immunohistochemical study using the tissue microarray technique. Romanian journal of morphology and embryology= Revue roumaine de morphologie et embryologie.

[18] Petrişor O, Giuşcă SE, Sajin MA, Dobrescu G, Căruntu ID (2008). Ki-67, p53 and bcl-2 analysis in colonic versus rectal adenocarcinoma. Rom J Morphol Embryol.

[19] Sharifi N, Ghaffarzadegan K, Ayatollahi H, Shakeri MT, Sadeghian MH, Azari JB (2009). Evaluation of angiogenesis in colorectal carcinoma by CD34 immunohistochemistry method and its correlation with clinicopathologic parameters. Acta Medica Iranica.

[20] Afrem G, Mogoantă SS, Secureanu FA, Olaru A, Neamţu C, Totolici BD (2011). Study of molecular prognostic factors Bcl-2 and EGFR in rectal mucinous carcinomas. Romanian journal of morphology and embryology.

[21] Watson AJM, Merritt AJ, Jones LS (1996). Evidence for reciprocity of bcl-2 and p53 expression in human colorectal adenomas and carcinomas. British Journal of Cancer.

